# Systemic Profiles of microRNAs, Redox Balance, and Inflammation in Lung Cancer Patients: Influence of COPD

**DOI:** 10.3390/biomedicines9101347

**Published:** 2021-09-29

**Authors:** Liyun Qin, Maria Guitart, Víctor Curull, Albert Sánchez-Font, Xavier Duran, Jun Tang, Mireia Admetlló, Esther Barreiro

**Affiliations:** 1 Pulmonology Department-Muscle Wasting and Cachexia in Chronic Respiratory Diseases and Lung Cancer Research Group, IMIM-Hospital del Mar, Parc de Salut Mar, Health and Experimental Sciences Department (CEXS), Universitat Pompeu Fabra (UPF), Universitat Autònoma de Barcelona, Parc de Recerca Biomèdica de Barcelona (PRBB), 08003 Barcelona, Spain; liyun.qin@e-campus.uab.cat (L.Q.); mguitart@imim.es (M.G.); VCURULL@parcdesalutmar.cat (V.C.); ASanchezF@parcdesalutmar.cat (A.S.-F.); jun.tang2@e-campus.uab.cat (J.T.); madmetllo@parcdesalutmar.cat (M.A.); 2 Centro de Investigación en Red de Enfermedades Respiratorias (CIBERES), Instituto de Salud Carlos III (ISCIII), 08003 Barcelona, Spain; 3 Scientific and Technical Department, Hospital del Mar-IMIM, 08003 Barcelona, Spain; xduran@imim.es

**Keywords:** LC and COPD, microRNAs, prooxidants and antioxidants, GSH, inflammatory cytokines, associations between clinical and biological variables

## Abstract

Lung cancer (LC) risk increases in patients with chronic respiratory diseases (COPD). MicroRNAs and redox imbalance are involved in lung tumorigenesis in COPD patients. Whether systemic alterations of those events may also take place in LC patients remains unknown. Our objectives were to assess the plasma levels of microRNAs, redox balance, and cytokines in LC patients with/without COPD. MicroRNAs (RT-PCR) involved in LC, oxidized DNA, MDA-protein adducts, GSH, TEAC, VEGF, and TGF-beta (ELISA) were quantified in plasma samples from non-LC controls (n = 45), LC-only patients (n = 32), and LC-COPD patients (n = 91). In LC-COPD patients compared to controls and LC-only, MDA-protein adduct levels increased, while those of GSH decreased, and two patterns of plasma microRNA were detected. In both LC patient groups, miR-451 expression was downregulated, while those of microRNA-let7c were upregulated, and levels of TEAC and TGF-beta increased compared to the controls. Correlations were found between clinical and biological variables. A differential expression profile of microRNAs was detected in patients with LC. Moreover, in LC patients with COPD, plasma oxidative stress levels increased, whereas those of GSH declined. Systemic oxidative and antioxidant markers are differentially expressed in LC patients with respiratory diseases, thus implying its contribution to the pathogenesis of tumorigenesis in these patients.

## 1. Introduction

Lung cancer (LC) is still a leading cause of cancer-related mortality worldwide. Several etiologic factors contribute to LC, among which chronic obstructive pulmonary disease (COPD) is a major contributor [1,2,3,4]. In patients with COPD, particularly in emphysema phenotype, LC development was five times greater than in smokers with no COPD [4,5]. Recently, our group and others have demonstrated the implications of relevant biological mechanisms in the increased LC predisposition seen in patients with COPD [6,7,8,9,10,11,12,13].

Adaptation to environmental factors and tumorigenesis are mediated through many different biological events including epigenetics. Epigenetic control of cellular processes includes DNA methylation, histone acetylation and methylation, and chromatin remodeling in tumor development and progression [14]. Furthermore, non-coding single-stranded RNA molecules (microRNAs) have also been shown to regulate the cellular processes involved in lung tumorigenesis, such as cell proliferation and invasion, apoptosis, angiogenesis, and adaptation to hypoxia [15,16,17]. Moreover, patterns of microRNA expression may also be used in clinics, given their prognosis value [16,18]. In a previous study from our group [13], expression levels of the microRNAs miR-21, miR-200b, miR-210, and miR-let7c were increased in lung tumor samples from non-small cell LC (NSCLC) patients with COPD compared to those with no COPD. Whether levels of the same microRNAs may be differentially expressed in the plasma of LC patients with and without COPD remains to be seen.

Increased oxidative stress and inflammatory events have also been shown to contribute to the greater predisposition of patients with COPD to develop lung tumors [19]. In this study [19], a differential expression profile of oxidative stress and inflammatory markers was found in the blood and tumors of patients with LC-COPD compared to patients with no COPD. Importantly, increased oxidative stress may also trigger the expression of several microRNAs under pathologic conditions [20].

Several cytokines and growth factors may also mediate lung tumorigenesis in patients with COPD [21,22]. Cellular processes such as apoptosis, repair, and angiogenesis can be mediated through the action of inflammatory molecules [21,22]. Furthermore, vascular endothelial growth factor (VEGF) and transforming growth factor (TGF)-beta have also been shown to promote lung tumorigenesis through increased tumor growth and metastasis in patients with underlying respiratory diseases [23,24,25].

Hence, we hypothesized that a differential expression profile of microRNAs known to be involved in lung tumorigenesis may be identified in the blood compartment of patients with LC and underlying COPD compared to those without COPD. Elucidation of those biological events may add insight into the mechanisms that render COPD patients more prone to develop LC. Moreover, associations with oxidative stress and inflammatory markers were also assessed. Thus, our objectives were that, in plasma samples from LC patients with and without COPD, the following mechanisms were explored: (1) expression levels of microRNAs known to be involved in lung carcinogenesis; (2) redox balance, prooxidant and antioxidant markers; (3) VEGF and TGF-beta 1 protein levels; (4) relationships between clinical and biological variables; and (5) potential associations between expression levels of microRNAs and those of oxidative stress and inflammatory cytokines. A group of non-LC control subjects was also included in the current investigation.

## 2. Methods

### 2.1. Study Subjects

This was a hospital-based study in which patients and control subjects were recruited consecutively for 10 years (2009–2019). For the investigation, 168 Caucasian patients were recruited in total. Specifically, 123 patients with NSCLC were recruited from the Lung Cancer Clinic of the Respiratory Medicine Department at Hospital del Mar (Barcelona, Spain). Ninety-one out of the 123 patients had underlying COPD and 32 patients had NSCLC with no COPD. A group of non-tumor control subjects (n = 45) were also recruited for the purpose of the investigation from the COPD Clinics at Hospital del Mar. Therefore, for the purpose of comparisons, the following study groups were established: (1) 45 subjects without LC (32 males, non-LC control group), (2) 32 patients with only LC (15 males, LC-only group), (3) 91 patients with LC and COPD (82 males, LC-COPD group). COPD was defined following existing guidelines [26,27]. Exclusion criteria for all the patients and control subjects included other chronic cardiovascular or respiratory disorders, chronic metabolic diseases, signs of severe bronchial inflammation and/or infection, current or recent invasive mechanical ventilation, chronic oxygen therapy, and poor tolerance and/or collaboration. Approval was obtained from the institutional Ethics Committee on Human Investigation (Hospital del Mar-IMIM, Barcelona, protocol # 2008/3390/I, 4 February 2008, following World Medical Association guidelines (Helsinki Declaration of 2008) for research on human beings. Informed written consent was obtained from all participants.

### 2.2. Clinical Assessment

Nutritional evaluation included the assessment of body mass index (BMI) and blood analytical parameters in all participants. Lung function parameters were determined in all study subjects following standard procedures [11,13,19]. TNM staging [28,29] was determined only in all patients with LC.

### 2.3. Blood Samples

In all the study patients and non-LC control subjects, blood samples were obtained from the arm vein after an overnight fasting period. Blood specimens were centrifuged at 1500× *g* for 15 min to collect the plasma samples, which were immediately frozen at −80 °C until further analyses.

### 2.4. Quantification of microRNAs

*RNA isolation*. Previously described methodologies were used for this set of experiments [13]. RNA was isolated from 500 microL plasma samples using 500 microL TRIzol reagent (Cat. 15596026, Thermo Fisher Scientific, Waltham, MA, USA). After incubation of the samples at room temperature for 10 min to achieve complete dissociation of nucleoprotein complexes, 200 microL chloroform were added, and samples were then centrifuged at 13,500 rpm at 4 °C for 15 min. The aqueous phase was recovered, and the RNA was precipitated with 600 microL isopropanol. Subsequently, samples were incubated at 4 °C for 30 min and were then cooled down to −20 °C overnight. After thawing the samples at room temperature, they were centrifuged at 13,500 rpm at 4 °C for 10 min, and the supernatant was removed. The remaining pellet was then washed using one mL solution of 75% ethanol to be subsequently centrifuged at 9000 rpm at 4 °C for five minutes. The RNA containing pellet was air-dried for 30 min and was then dissolved in 20 microL RNase-free water. To assess the quality and purity of the isolated RNA, concentrations of total RNA were determined using NanoDrop 1000 (Thermo Fisher Scientific, Waltham, MA, USA) according to the manufacturer’s instructions.

*MicroRNA reverse transcription (RT).* TaqMan^®^ Advanced miRNA cDNA Synthesis Kit (Cat. A28007, Thermo Fisher Scientific, Waltham, MA, USA) was used to prepare cDNA templates following the manufacturer’s instructions. Total RNA isolated samples were manipulated to add a poly (A) tailing on the 3′ position and an adaptor on the 5′ position of the mature microRNAs. Initially, 3 microL Poly (A) reaction mix (0.5 microL Poly (A) buffer, 0.5 microL ATP, 0.3 microL Poly (A) enzyme, and 1.7 microL RNase-free water) was mixed with 2 microL of each sample. The mixture was then incubated in a thermal cycler (Geneamp PCR System 2400, Perkin Elmer, Waltham, MA, USA) to perform the polyadenylation reaction at 37 °C for 45 min. This step was followed by incubation at 65 °C for 10 min to stop the reaction. Immediately, the samples were supplemented with 10 microL ligation reaction mix (3 microL ligase buffer, 4.5 microL PEG 8000, 0.6 microL ligation adaptor, 1.5 microL RNA ligase, and 0.4 microL of RNase-free water) to incorporate the adaptor at the 5′ position. Samples underwent standard cycling at 16 °C for 60 min. MicroRNAs with both poly (A) tail and the adaptor were reverse transcribed (RT) to achieve cDNA. First, modified microRNA samples were mixed with 15 microL RT reaction mix (6 microL RT buffer, 1.2 microL dNTP mix, 1.5 microL universal RT primer, 3 microL RT enzyme mix, and 3.3 microL RNase-free water). Samples were subsequently incubated in the thermal cycler at 42 °C for 15 min to perform the reverse transcription, and finally, they were incubated at 85 °C for 5 min to stop the reaction.

A cDNA amplification step was performed to increase the number of cDNA molecules. Forty-five microL of miR-Amp reaction mix (25 microL miR-Amp master mix, 2.5 microL miR-Amp primer mix, and 17.5 microL RNase-free water) from the synthesis kit were mixed with 5 microL RT reaction product. The amplification reaction consisted of different cycles: enzyme activation at 95 °C for 5 min, denaturation at 95 °C for three seconds, and finally, the extension of the cDNA at 60 °C for 30 s. Denaturation and extension cycles were repeated 14 more times to ensure a sufficient quantity of cDNA. Subsequently, samples were incubated at 99 °C for 10 min to stop the reaction, and they were finally kept at −80 °C up until the performance of the real-time polymerase chain reaction (PCR) procedures.

*Quantitative real time-PCR amplification (qRT-PCR)*. Real-time PCR was performed using specific primers for the target microRNAs in the study: miR-451, miR-210, miR-126, miR-21, miR-let7c, miR-145, miR-200b, and miR-223 (Table 1). Taqman advanced microRNA 159a assay from *Arabidopsis thaliana* was used as an exogenous control in order to normalize the miRNA amplification. Briefly, five microL of the resulting cDNA samples were mixed with one microL of each specific primer, 10 microL TaqMan fast-advanced master mix (Cat. 4444964, Thermo Fisher Scientific), and four microL RNase-free water. The samples were run in a thermal cycler (QuantStudio system, Thermo Fisher Scientific). The first step was the enzyme activation, achieved at 95 °C for 20 s, which was followed by 40 combined cycles of denaturation (95 °C for one second) and final annealing (60 °C for 20 s). Duplicates from all samples were run, and the average value was calculated for all the study samples. The results obtained from the experiments were collected and analyzed using the ExpressionSuite Software version 1.1 from Applied Biosystems (ThermoFisher Scientific), in which the comparative C_T_ method (2^−ΔΔCT^) for relative quantification was used [30].

### 2.5. Quantification of Oxidative Stress Markers and Cytokines

In a subset of representative individuals: 40 non-LC control subjects, 19 LC-only patients, and 20 LC-COPD patients, markers of oxidative stress and cytokines were also analyzed in the blood samples. 

*Oxidatively damaged DNA*. Levels of oxidative DNA adduct 8-hydroxy-2-deoxy guanosine (8-OHdG) were measured in plasma using the DNA Damage (8-OHdG) ELISA kit (StressMarq Biosciences _INC_., Victoria, BC, Canada) following the specific manufacturer’s instructions and previously described methodologies [13,19]. Briefly, 50 microL of plasma was incubated with 50 microL of antibody per well at room temperature for one hour in a plate cover. After several washes, samples were incubated with 3,3′,5,5′-tetramethylbenzidine (TMB) substrate in the dark at room temperature for 30 min. Immediately afterwards, 100 microL of stop solution was poured into each well. Samples were then shaken from side to side and thoroughly mixed with the solution. After terminating this reaction, the absorbance was read at 450 nm in all the sample wells. A standard curve was always generated with each assay run. Intra-assay coefficients for all the samples ranged from 0.17% to 9.80%. The minimum detectable concentration of DNA in plasma was set to be 0.94 ng/mL (StressMarq Biosciences _INC_, Victoria, BC, Canada).

*Malondialdehyde (MDA)-protein adducts.* Levels of MDA-protein adducts were measured in plasma using the OxiSelect^TM^ MDA Adduct Competitive ELISA Kit (Cell Biolabs, Inc., San Diego, CA, USA) following the specific manufacturer’s instructions and previously described methodologies [13,19]. First, an MDA conjugate was coated on an ELISA plate, then 50 microL of plasma specimens were added to the MDA conjugate preabsorbed ELISA plate and incubated at room temperature for 10 min on an orbital shaker. After a brief incubation, the primary antibody was added and incubated at room temperature for one hour on an orbital shaker. After three washes, samples were incubated with secondary antibody at room temperature for another hour on an orbital shaker. After three washes, the substrate solution was added at room temperature for 20 min, and samples were again shaken on an orbital shaker. Immediately afterwards, 100 microL of the stop solution was poured into each well. Samples were then thoroughly mixed with the solution. After completing this reaction, the absorbances were read at 450 nm. A standard curve was always generated with each assay run. Intra-assay coefficients of variation for all the samples ranged from 0.11% to 9.73%. The minimum detectable concentration of MDA-protein adducts in plasma was set to be 6 pmol/mL (Cell Biolabs, Inc., San Diego, CA, USA).

*Reduced glutathione (GSH)*. GSH was measured in the blood using the Human reduced glutathione (GSH) ELISA Kit (MyBioSource, San Diego, CA, USA) following the specific manufacturer’s instructions and previously described methodologies [11,19]. Fifty microL samples were added to every sample well and incubated with horseradish (HRP)-conjugate reagent at 37 °C for 60 min. The plate was covered with a closure plate membrane during the experiment. After four washes, 50 microL chromogen solution A and 50 microL chromogen solution B were added to each well, and samples were then incubated at 37 °C in the dark for 15 min. Finally, 50 microL of the stop solution were poured into each well. The absorbance in each sample was read at 450 nm. Intra-assay coefficients for all the samples ranged from 0.15% to 9.93%. The minimum detectable concentration of GSH in plasma was set to be 1.56 µmol/L (MyBioSource, San Diego, CA, USA).

*Plasma levels of Trolox Equivalent Antioxidant Capacity (TEAC)*. TEAC levels were determined using the OxiSelect^TM^ Trolox Equivalent Antioxidant Capacity (TEAC Assay Kit (ABTS, Cell Biolabs, Inc., San Diego, CA, USA) following the manufacturer’s instructions. Twenty-five microL samples were added to the microplate well, and upon addition of 150 microL of the diluted 2,2′-azino-bis (3-ethylbenzothiazoline-6-sulfonic acid) reagent, samples were mixed thoroughly. Samples were then incubated on an orbital shaker for five minutes. Finally, the absorbance was read at 405 nm in all the sample wells. Antioxidant activity was determined by comparison with the Trolox standards. Intra-assay coefficients of variation for all the samples ranged from 0.03% to 9.75%. (Cell Biolabs, Inc., San Diego, CA, USA). The minimum detectable concentration of TEAC in plasma was set to be 250.29 g/mol (Cell Biolabs, Inc., San Diego, CA, USA).

*Cytokines*. Protein levels of the cytokines TGF-beta 1 and VEGF-A were quantified using specific ELISA kits (RayBiotech, Norcross, GA, USA) for each cytokine following the manufacturer’s instructions and previously described methodologies [13,31,32]. All samples were incubated with the specific primary antibodies and were always run together in each assay. Before commencing the assay, samples and reagents were equilibrated to room temperature. Standards (100 microL) were performed as per the manufacturer’s instructions. The protocol was followed according to the corresponding manufacturer’s instructions for each cytokine. Intra-assay coefficients of variation for all the samples ranged from 0.13% to 9.66% (TFG-beta1) and from 0.13% to 9.93% (VEGF). Absorbances were read at 450 nm in all sample wells. A standard curve was always generated with each assay run. The minimum detectable concentration of TGF-beta 1 was 18 pg/mL (RayBiotech, Norcross, GA, USA). The minimum detectable concentration of VEGF-A was 3.59 pg/mL (RayBiotech, Norcross, GA, USA).

### 2.6. Statistical Analysis

Normality of the study variables was tested using the Shapiro–Wilk test. Data are expressed as mean and standard deviation (SD) in tables and figures. MicroRNA-451 was selected as the target variable to calculate the sample size. Having a priori unbalanced design, where the LC-COPD group had twice as many patients as the LC group and the control group, a minimum sample of 27 non-LC controls, 27 LC, and 54 LC-COPD patients was required to achieve an 80% statistical power, taking a within groups mean square error equals to 4393. Statistical significance was established at *p* < 0.05. Potential differences of quantitative variables among the study groups were assessed using the one-way analysis of variance (ANOVA) and Tukey’s post hoc analysis to adjust for multiple comparisons. Chi-square test was employed to assess potential differences in categorical variables (smoking history) among the three study groups. Comparisons of the results obtained from the micro-RNA expression analyses within each study group were determined using the Duncan multiple comparison test. Correlations between clinical and biological variables were explored using the Pearson’s correlation coefficient. Bivariate analysis was performed to test associations between two variables for the study population. All statistical analyses were performed using the software SPSS 23.0 (SPSS Inc, Chicago, IL, USA).

## 3. Results

### 3.1. Clinical Characteristics

Anthropometric variables such as age, body weight, and BMI did not significantly differ among the study subjects (Table 2). The proportions of active smokers were similar in the three study groups (Table 2). However, the proportions of ex-smokers were significantly greater in the LC-COPD group than in the LC-only group and showed a tendency to be higher than in the control subjects (*p* = 0.1, Table 2). The proportions of never smokers were significantly lower in the LC-COPD patients than in the LC-only and the control subjects (Table 2). No significant differences were seen in the number of packs-year among the study groups (Table 2). LC-COPD patients had moderate airway obstruction compared to LC-only and the controls, who had normal lung function parameters (Table 2). LC staging was similar between the two study groups (Table 2). In both groups of patients, nutritional status was preserved, and globular sedimentation velocity (GSV) was increased compared to the controls (Table 2). For both groups of patients, the comorbidities and different treatments are illustrated in Table 2. No significant correlations were found between any of the comorbidities and treatments with the study biological markers.

### 3.2. Differential Pattern of Systemic microRNA Expression in LC Patients

**Figure 1:**
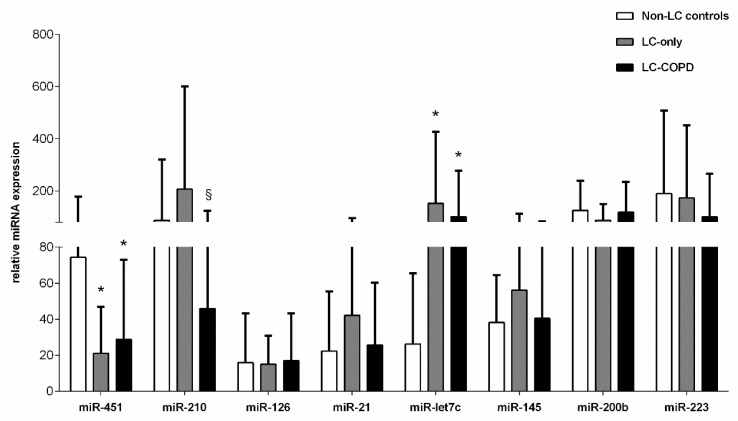
Mean values and standard deviation (relative expression) of microRNA (miR) expression in plasma samples of non-LC controls (white bars), LC-only patients (grey bars), and LC-COPD patients (black bars). Statistical significance: * *p* < 0.05 between either of the two groups of patients with LC and the non-LC control subjects; ^§^ *p* < 0.05 for comparisons between LC-only patients and LC-COPD patients. For the sake of clarity, the absence of statistical symbols indicates that no significant differences were found between groups for the different study comparisons.

Compared to the non-LC controls, the plasma levels of miR-451 significantly decreased in both groups of patients (Figure 1). Systemic levels of miR-210 significantly declined in LC-COPD compared to LC-only patients (Figure 1). The plasma levels of miR-let7c were significantly greater in LC-only and LC-COPD patients than in the control subjects (Figure 1). Significant associations were detected between plasma microRNA-let7c expression levels and total leukocytes and neutrophil counts (r = 0.467, *p* = 0.011, r = 0.498, and *p* = 0.006, respectively). No significant differences were seen in the plasma expression levels of miR-126, miR-21, miR-145, miR-200b, or miR-223 among the study groups (Figure 1).

**Figure 2:**
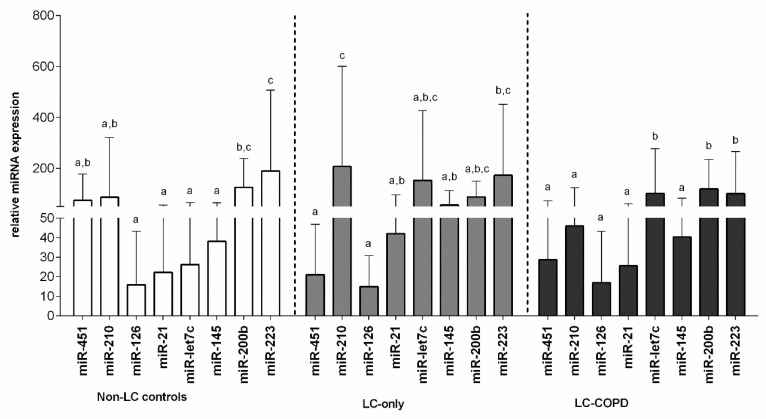
Mean values and standard deviation (relative expression) of microRNA (miR) expression in plasma samples of non-LC controls (white bars), LC-only patients (grey bars), and LC-COPD patients (black bars). Statistical analyses were performed separately for each study group of subjects. The letters a,b,c indicate the statistical significance: the same letter indicate no statistically significant difference among the groups for a given microRNA. In each study group, the expression levels of the study microRNAs did not differ among them if they shared the same letter. Different letters reflect different levels of expression for each study group.

In the non-LC control group, the patterns of expression of miR-126, miR-21, miR-let7c, and miR-145 were similar, whereas miR-451, miR-210, and miR-200b shared an intermediate pattern and miR-223 followed a different pattern (Figure 2). In the LC-only group, miR-451 and miR-126 followed an identical pattern of expression, while miR-21, miR-let7c, miR-145, miR-200b, and miR-223 followed an intermediate pattern, and miR-210 expressed a different pattern (Figure 2). In the LC-COPD group, plasma expression levels of miR-451, miR-210, miR-126, miR-21, and miR-145 were similar, whereas expression levels of miR-let7c, miR-200b, and miR-223 followed a completely different pattern of expression (Figure 2).

### 3.3. Redox Balance in LC Patients

#### 3.3.1. Oxidative Stress Markers

**Figure 3:**
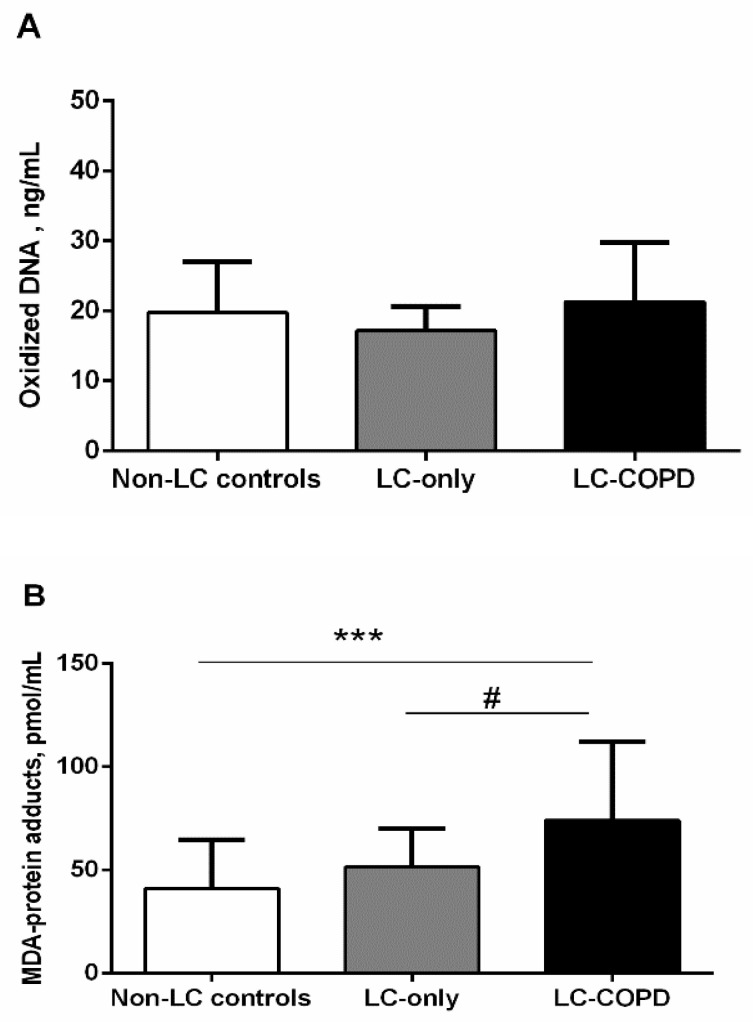
(**A**) Mean values and standard deviation of levels of oxidized DNA (ng/mL) did not differ between the study groups of subjects. (**B**) Mean values and standard deviation of level of MDA-protein adducts (pmol/mL) were significantly higher in LC-COPD patients compared to non-LC control subjects and LC-only patients. Statistical significance is as follows: *** *p* < 0.001 between LC-COPD patients and the non-LC control subjects, # *p* < 0.05 between LC-COPD patients and LC-only patients. The absence of statistical symbols indicates that no significant differences were found between groups for the different study comparisons.

Levels of oxidized DNA did not significantly differ between the study groups (Figure 3A). MDA-protein adduct levels were significantly higher in LC-COPD patients compared to LC-only patients and non-LC controls (Figure 3B). No significant differences were seen in MDA-protein adduct levels between LC-only patients and the control subjects (Figure 3B).

#### 3.3.2. Antioxidants

**Figure 4:**
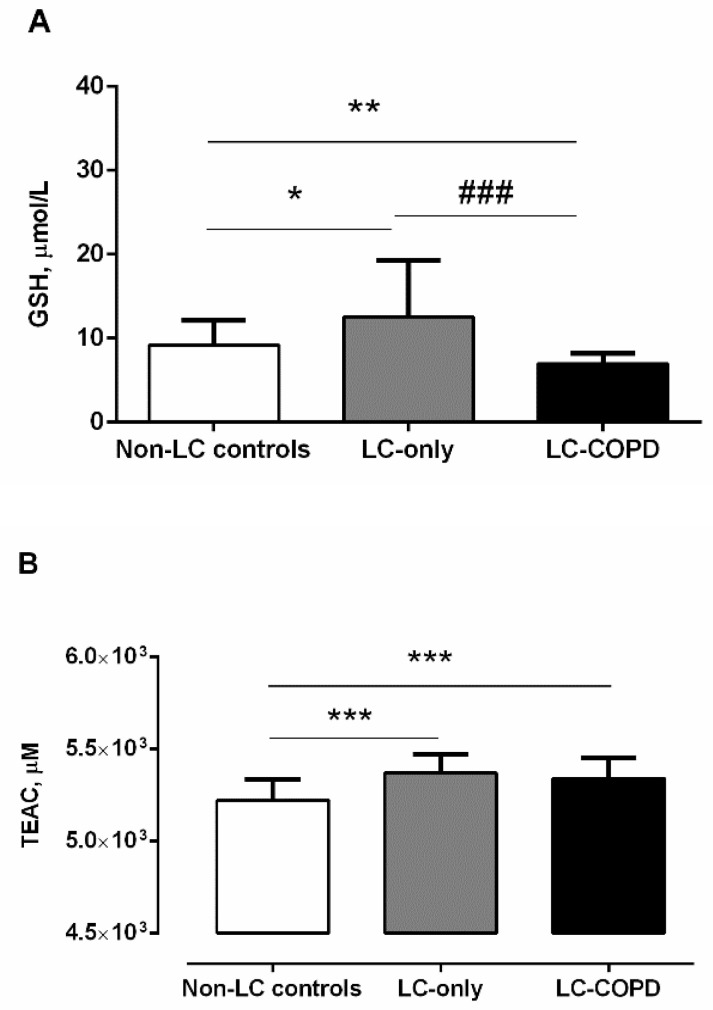
(**A**) Mean values and standard deviation of plasma GSH levels (µmol/L) were significantly higher in LC-only patients than in non-LC patients. However, levels of GSH in LC-COPD patients were significantly lower than in both non-LC controls and LC-only patients. Statistical significance: * *p* < 0.05, ** *p* < 0.01 between either of the two groups of patients with LC and the non-LC control subjects, and ^###^ *p* < 0.001 between LC-COPD and LC-only patients. (**B**) Mean values and standard deviation of plasma TEAC (µM) were significantly higher in both LC-COPD and LC-only patients compared to non-LC controls. Statistical significance is as follows: *** *p* < 0.001 between any group of LC patients and the non-LC controls. The absence of statistical symbols indicates that no significant differences were found between groups for the different study comparisons.

GSH levels were significantly higher in LC-only patients than in non-LC controls, whereas, in LC-COPD patients, the levels significantly declined compared to LC-only patients and the non-LC controls (Figure 4A). A significant positive correlation (r = 0.513 and *p* = 0.001) was detected between plasma GSH levels and FEV_1_/FVC among all the patients. Additionally, among LC-COPD patients, total neutrophil counts were almost inversely associated with GSH plasma levels (r = −0.421 and *p* = 0.082). TEAC levels were significantly greater in both LC-COPD and LC-only patients compared to non-LC controls (Figure 4B). No significant differences were detected in TEAC levels between the two LC patient groups (Figure 4B).

### 3.4. Cytokines in LC Patients

**Figure 5:**
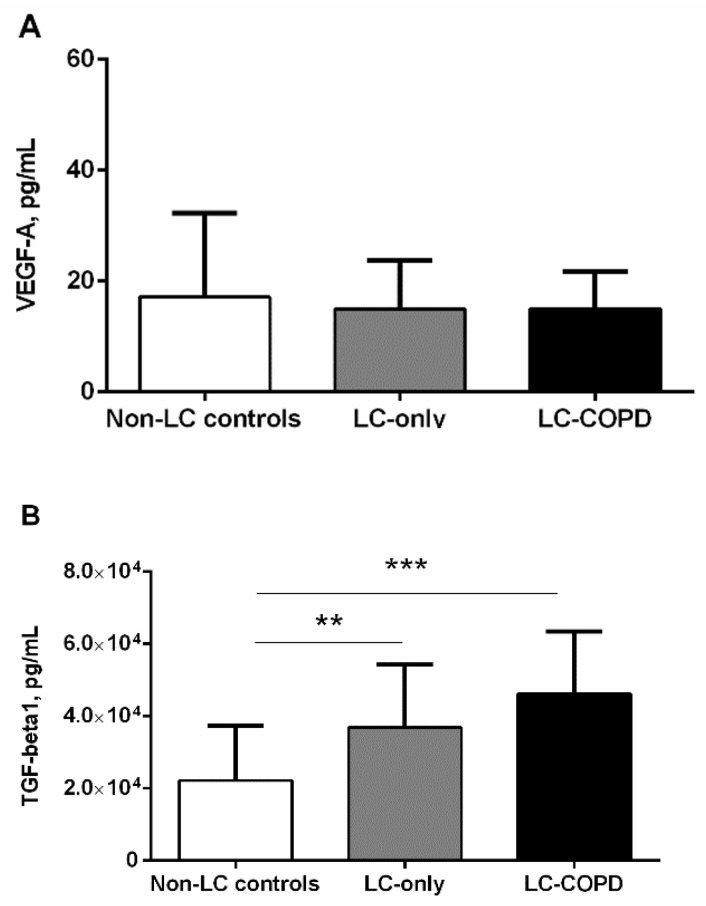
(**A**) Mean values and standard deviation of plasma VEGF-A levels (pg/mL) did not significantly differ between the study groups. (**B**) Mean values and standard deviation of plasma TGF-beta1 were significantly greater in both LC-COPD and LC-only patients compared to non-LC control subjects. Statistical Significance: ** *p* < 0.01, *** *p* < 0.001 between any group of LC patients and the non-LC control subjects. The absence of statistical symbols indicates that no significant differences were encountered between groups for the different study comparisons.

Levels of VEGF-A did not significantly differ between the study groups (Figure 5A). Importantly, both groups of LC patients, especially those with COPD, exhibited a significant rise in plasma TGF-beta 1 levels compared to the non-LC control subjects (Figure 5B). No significant differences in TGF-beta 1 levels were detected between the two patient groups (Figure 5B). An almost significant positive correlation was observed between plasma TGF-beta 1 and microRNA-let7c expression levels (r = 0.354 and *p* = 0.064). Furthermore, significant inverse associations were observed between plasma TGF-beta 1 levels and DL_CO_ and K_CO_ among all the LC patients (r = −0.379 and *p* = 0.027 and r = −0.608 and *p* < 0.001, respectively).

### 3.5. Bivariate Analysis

The bivariate analysis showed that, in patients with LC-COPD, smoking history, except for current smokers, significantly differed from that reported in the LC patients (Table 3). Additionally, the bivariate analysis demonstrated that lung airflow limitation and diffusion capacity were significantly impaired in patients with LC-COPD compared to LC patients (Table 3). Furthermore, levels of the blood parameters albumin, miR-210, and the antioxidant GSH were significantly reduced in the LC-COPD compared to LC patients, while those of MDA-protein adducts were significantly higher in the former patients than in the latter group (Table 3).

## 4. Discussion

In the current investigation, the most relevant findings were that, in LC-COPD, two different patterns of plasma microRNA expression were observed. Moreover, expression of miR-451 was significantly downregulated in both groups of patients with LC compared to non-LC controls. Plasma protein oxidation levels, as measured by MDA-protein adducts, were increased only in patients with LC-COPD compared to non-LC controls. Nonetheless, systemic levels of the antioxidant GSH significantly declined in LC-COPD patients compared to both LC-only patients and the non-LC control subjects. Conversely, a significant rise in plasma antioxidant TEAC levels was detected in both groups of LC patients compared to the controls. In this study, blood levels of VEGF did not vary across groups, whereas TGF-beta plasma levels significantly increased in both groups of LC patients compared to non-LC controls. The most relevant results obtained in the study are discussed below.

Importantly, systemic levels of microRNA-451 were reduced in both groups of patients with LC, and COPD did not significantly influence those levels. Low levels of the tumor-suppressive miR-451 were associated with poor prognosis in NSCLC patients [33]. Additionally, the tumor suppressor miR-451 was also shown to enhance cisplatin sensitivity via regulation of Mcl-1 expression, suggesting that novel therapeutic targets may be designed thereafter [34,35]. In a previous study from our group [13], levels of miR-451 were also reduced in the tumors of patients with LC, particularly in those with COPD. Hence, miR-451 may be a surrogate of lung tumorigenesis that could help monitor patients in the clinics.

Interestingly, expression levels of miR-let7c were upregulated in plasma samples of both groups of LC patients compared to the non-LC controls. Furthermore, significant associations were found between plasma microRNA-let7c expression levels and inflammatory cell counts, which were increased in both groups of LC patients. These are novel findings that deserve further attention. The results encountered in the current study are in line with those previously reported in the lung tumors of patients with LC, especially in those with COPD [13]. In that study [13], levels of the *k-RAS* gene were also downregulated in the lung tumors of the LC patients with underlying COPD. It would be possible to conclude that, as miR-let7c acts a tumor suppressor in cancer cells, underlying respiratory conditions, such as in COPD, may have induced a positive feedback loop to counterbalance the deleterious effects of cancer biology. In keeping with this, miR-let7c expression levels negatively correlated with metastasis, vascular invasion, and poor survival in NSCLC patients, whose miR-let7c levels were downregulated [36]. Whether patients also had a concomitant respiratory disease was not explored in that study [36]. Moreover, it was also demonstrated that the upregulation of miR-let7c was probably involved in the chemoresistance of lung cancer in patients [37]. Another finding that deserves attention is the significant decrease in miR-210 levels that was observed in the LC-COPD patients compared to LC patients, as confirmed in the bivariate analysis. As miR-210 is upregulated during hypoxia [38], it may be a useful marker of tumor development in patients with underlying COPD. Future research should be targeted at exploring the potential role of miR-210 in the lung predisposition of COPD. Thus, miR-210 may be used as a surrogate marker to monitor LC predisposition in patients with COPD.

Oxidative stress favors carcinogenesis as a result of the processes involved in neoplastic transformation and DNA mutations [22]. Posttranslational modifications induced by oxidative damage of proteins, lipids, and DNA promote the cell viability and growth of cancer cells [12]. In fact, proteins, DNA, and lipids are major targets for the action of oxidants that are not counterbalanced by the tissue antioxidant capacity, leading to the development of oxidative stress [39,40,41]. Reactive carbonyl derivatives (aldehydes and ketones) are formed by the reaction of oxidants with several amino acid residues (e.g., lysine, proline, and threonine). On the other hand, Michael-addition reactions of lysine, cysteine, or histidine residues with α,ß-unsaturated aldehydes (e.g., malondialdehyde, MDA) may also lead to the formation of reactive carbonyls resulting from the peroxidation of polyunsaturated fatty acids of the membranes [42,43,44]. In the current investigation, MDA-protein adduct plasma levels were significantly greater in the LC-COPD patients compared to both non-LC control subjects and the LC-only patients. These findings are in line with those previously reported [11], in which MDA-protein adduct levels were increased in the lung tumors of patients with LC-COPD compared to LC without the underlying respiratory condition. These results were also confirmed in the bivariate analysis. Taken together, these results suggest that COPD per se may induce the rise in systemic oxidative stress levels in LC patients. As systemic oxidative stress levels have also been reported to be increased in patients with only COPD [39,45], this is a likely explanation. Collectively, these results suggest that oxidative stress markers should be detected regularly in the clinics as an early marker of lung tumorigenesis, particularly in patients with chronic respiratory diseases such as COPD.

Powerful antioxidant systems protect cells from oxidatively induced damage. As such, superoxide dismutase isoforms, catalase, and glutathione peroxidases are counted among the most abundant antioxidant enzymes. Non-enzymatic antioxidant systems complement the action of antioxidant enzymes within cells. The most abundant non-protein thiol glutathione is a water-soluble compound, which is widely distributed within tissues. Levels of reduced glutathione (GSH) indicate the redox potential of a tissue. In the current investigation, GSH plasma levels were significantly lower in the LC-COPD patients than in both LC-only patients and the non-LC controls. These results were also confirmed in the bivariate analysis. Additionally, the degree of airway obstruction as measured by FEV_1_/FVC significantly correlated with plasma GSH levels, suggesting that patients with greater airflow limitation were those with lower levels of GSH. Moreover, neutrophil counts were inversely associated with GSH in this study. Taken together, these relevant findings reveal that the reduction in redox potential observed in the patients with underlying COPD may predispose them to develop LC. In fact, a recent meta-analysis has put the line forward that GSH plasma levels were reduced in patients with COPD, suggesting that this mechanism is likely involved in the pathogenesis of the chronic airway disease and could also be part of the greater predisposition of these patients to develop LC [45]. Taken together, these findings imply that GSH may be useful for monitoring the lung tumorigenesis process in patients with COPD. In LC patients with no COPD, however, plasma levels of GSH were increased compared to non-LC control subjects. Modifications in redox balance including antioxidant levels were also shown to be part of the pathophysiology of several cancer types, including LC. Interestingly, a significant rise in plasma levels of TEAC were observed in both groups of LC patients. As far as we are concerned, these are novel results, implying that the antioxidant capacity relative to the standard Trolox (vitamin E analog) was increased in response to lung carcinogenesis among all the patients irrespective of COPD.

Inflammatory cytokines such as VEGF and TGF-beta have been demonstrated to participate in the pathophysiology of LC development in patients with chronic respiratory diseases [21,22]. Cell mechanisms such as proliferation and repair, apoptosis, and angiogenesis may be hindered by increased levels of several interleukins and cytokines [21,22]. In the present study, the plasma levels of VEGF did not vary between the study groups, while a significant rise in plasma TGF-beta levels was detected in both groups of LC patients. Importantly, significant inverse associations were observed between plasma TGF-beta levels and diffusion capacity among all the LC patients. These findings suggest that patients with a certain degree of emphysema were those exhibiting greater TGF-beta plasma levels, despite the fact that these levels did not differ between the two patient groups. Indeed, similar findings were previously reported in patients with LC with and without COPD [21,22]. In a recent meta-analysis [46], TGF-beta was shown to help predict the worse prognosis in patients with LC, independently of the presence of underlying respiratory diseases. On this basis, it may be possible to conclude that TGF-beta can be used as a prognosis marker in the follow-up of patients with LC. Nonetheless, TGF-beta does not seem to help predict lung tumorigenesis in patients with underlying COPD in the clinics.

## 5. Study Limitations

In the current investigation, the number of patients and controls analyzed using ELISA was smaller compared to those used in the microRNA analyses. The objective was to analyze all the samples synchronically within the same plate in order to minimize variability. The most representative subjects in each group, on the basis of the mRNA amplification during RT-PCR experiments, were selected for the purpose of this study. Despite these concerns, the study hypothesis has been confirmed and the sample size calculations were correct in the investigation, as described in the Methods section.

## 6. Conclusions

A differential expression profile of microRNAs was detected in patients with LC, specifically of miR-451, miR-let7c, and miR-210. Furthermore, in LC patients with COPD, plasma oxidative stress levels (MDA-protein adducts) increased, whereas those of the powerful antioxidant GSH declined. Redox imbalance is differentially expressed in LC patients with underlying respiratory diseases, which reveal its potential implications in the pathogenesis of tumorigenesis in these patients. Decreased levels of the antioxidant GSH may be used as a surrogate biomarker of lung tumorigenesis in patients with chronic respiratory diseases in the clinics. These findings have clinical implications in the management and monitoring of patients with LC, with a special focus on those with underlying COPD.

## Figures and Tables

**Table 1:** MicroRNA assays used for the quantitative analyses of the target genes using real-time PCR.

Assay Name	Assay ID	miRbase Accession Number
hsa-miR-451a	478107_mir	MIMAT0001631
hsa-miR-210-3p	477970_mir	MIMAT0000267
hsa-miR-126-3p	477887_mir	MIMAT0000445
hsa-miR-21-5p	477975_mir	MIMAT0000076
hsa-let7c-5p	478577_mir	MIMAT0000064
hsa-miR-145-5p	477916_mir	MIMAT0000437
hsa-miR-200b-3p	477963_mir	MIMAT0000318
hsa-miR-223-3p	477983_mir	MIMAT0000280
ath-miR159a	478411_mir	MIMAT0000177

Abbreviations: ID, identification; hsa, homo sapiens; miR, microRNA; MIMAT, mature microRNA; ath, arabidopsis thaliana.

**Table 2:** Clinical characteristics of the study patients.

	Non-LC Controls	LC-Only	LC-COPD
	N = 45	N = 32	N = 91
Anthropometry			
Age (years)	63 (10)	64 (11)	67 (9)
Male, N/female, N	32/13	15/17	82/9
Body weight (kg)	75 (16)	70 (14)	72 (12)
BMI (kg/m^2^)	27 (5)	26 (4)	25 (4)
Smoking history			
Active, N (%)	15 (33)	11 (34)	39 (43)
Ex-smoker, N (%)	17 (38)	9 (28)	48 (53) ^#^
Never smoker, N (%)	13 (29)	12 (38)	4 (4) ***^,###^
Packs-year	47 (26)	46 (15)	58 (21)
Lung function testing			
FEV_1_, % predicted	80 (24)	94 (15) ***	62 (14) ***^,###^
FEV_1_/FVC, % predicted	70 (12)	78 (6) ***	61 (9) ***^,###^
DL_CO_, % predicted	81 (22)	82 (19)	68 (19) **^,##^
K_CO_, % predicted	82 (22)	82 (15)	75 (21)
TNM staging			
Stage 0-II: N (%)	NA	20 (62)	53 (58)
Stage III: N (%)	NA	7 (22)	19 (21)
Stage IV: N (%)	NA	5 (16)	19 (21)
Blood parameters			
Total leukocytes/µL	8.2 (4.1) × 10^3^	10.5 (4.2) × 10^3^ *	9.7 (3.6) × 10^3^
Total neutrophils/µL	5.3 (2.8) × 10^3^	8.3 (4.4) × 10^3^ ***	7.2 (3.5) × 10^3^ *
Total lymphocytes/µL	2.1 (1.1) × 10^3^	1.4 (0.6) × 10^3^	1.9 (3.0) × 10^3^
Albumin (g/dL)	4.4 (0.6)	4.4 (0.9)	4.0 (0.6) **^,#^
Total proteins (g/dL)	7.3 (0.8)	7.0 (1.0)	7.1 (0.8)
CRP (mg/dL)	4.1 (8.2)	5.3 (9.4)	5.7 (7.8)
Fibrinogen (mg/dL)	451 (147)	463 (131)	481 (157)
GSV (mm/h)	16.3 (13.9)	27.6 (15.3) *	33.4 (22.0) **
Comorbidities			
Hypertension, N (%)	NA	10 (31.3)	45 (49.5)
Type 2 Diabetes mellitus, N (%)	NA	2 (6.3)	17 (18.7)
Dyslipidemia, N (%)	NA	2 (6.3)	26 (28.6)
Treatments			
Diuretic, N (%)	NA	5 (15.6)	20 (22.2)
Angiotensin converting enzyme inhibitors, N (%)	NA	2 (6.3)	6 (28.6)
Angiotensin-2 receptor blockers, N (%)	NA	2 (6.3)	14 (15.4)
Beta blockers, N (%)	NA	3 (9.4)	5 (5.5)
Calcium channel blockers, N (%)	NA	2 (6.3)	7 (7.7)
HMG-CoA-reductase, N (%)	NA	2 (6.3)	17 (18.7)
Biguanides, N (%)	NA	2 (6.3)	26 (28.6)
LAMA, N (%)	NA	NA	60 (65.9)
LABA, N (%)	NA	NA	30 (33)
Inhaled corticosteroids, N (%)	NA	NA	17 (18.7)

Values are expressed as mean (standard deviation). Abbreviations: COPD, chronic obstructive pulmonary disease; N, number of patients; m, meters; BMI, body mass index; FEV_1_, forced expiratory volume in one second; DLco, carbon monoxide transfer; K_CO_, Krough transfer factor; g, grams; TNM, tumor, nodes, metastasis; NA, not applicable; dL, deciliter; mg, milligrams; CRP, C-reactive protein; GSV, globular sedimentation velocity; LAMA, long-acting muscarinic antagonists; LABA, long-acting beta-agonists; mm, millimeters; h, hour. Statistical significance: * *p* < 0.05, ** *p* < 0.01, *** *p* < 0.001 between any study group compared to non-LC controls; ^#^ *p* < 0.05; ^##^ *p* < 0.01; ^###^ *p* < 0.001 between LC-COPD group compared to LC-only group.

**Table 3:** Bivariate analysis of clinical and biological variables in patients with LC with and without COPD.

	LC-Only	LC-COPD	*p* Value
Smoking history			
Never smokers, N (%)	12 (38)	4 (4)	<0.001
Ex-smokers, N (%)	9 (28)	48 (53)	0.023
Current smokers, N (%)	11 (34)	39 (43)	0.414
Packs-year, $\bar{x}$ (SD)	46 (15)	58 (21)	0.015
FEV_1_, %, $\bar{x}$ (SD)	94 (15)	62 (14)	<0.001
FEV_1_/FVC, $\bar{x}$ (SD)	78 (6)	61 (9)	<0.001
DL_CO_, %, $\bar{x}$ (SD)	82 (19)	68 (19)	0.001
Albumin, $\bar{x}$ (SD)	4.4 (0.9)	4.0 (0.6)	0.016
miR-210, $\bar{x}$ (SD)	207.13 (393.11)	45.95 (78.07)	0.006
MDA-protein adducts, $\bar{x}$ (SD)	51.52 (18.53)	74.20 (37.97)	0.033
GSH, $\bar{x}$ (SD)	12.54 (6.17)	6.97 (1.24)	0.001

Values are expressed as mean (standard deviation). Abbreviations: N, number of patients; FEV_1_, forced expiratory volume in one second; FVC, forced vital capacity; DLco, carbon monoxide transfer; MDA-protein adducts, malondialdehyde-protein adducts; GSH, reduced glutathione.

## Data Availability

The datasets are available from the corresponding authors upon reasonable request.
